# Large Language Model Recommendations for Empiric Antibiotics Versus Clinician Prescribing: A Non-Interventional Paired Retrospective Antimicrobial Stewardship Analysis

**DOI:** 10.3390/antibiotics15040368

**Published:** 2026-04-02

**Authors:** Ninel Iacobus Antonie, Vlad Alexandru Ionescu, Gina Gheorghe, Loredana-Crista Tiucă, Camelia Cristina Diaconu

**Affiliations:** 1 Faculty of Medicine, University of Medicine and Pharmacy Carol Davila Bucharest, 050474 Bucharest, Romania; vladalexandru.ionescu92@gmail.com (V.A.I.); gheorghe_gina2000@yahoo.com (G.G.); laslocrista@gmail.com (L.-C.T.); camelia.diaconu@umfcd.ro (C.C.D.); 2 Internal Medicine Department, Clinical Emergency Hospital of Bucharest, 105402 Bucharest, Romania; 3 Section of Medical Sciences, Academy of Romanian Scientists, 050045 Bucharest, Romania

**Keywords:** antimicrobial stewardship, antimicrobial resistance, large language model, paired retrospective study, OpenAI API, artificial intelligence

## Abstract

**Background/Objectives**: Antimicrobial resistance (AMR) remains a major global health threat, strengthening the case for antimicrobial stewardship strategies that limit unnecessary broad-spectrum empiric therapy while preserving timely escalation when clinically warranted. Before any clinical deployment of large language model (LLM)-based antibiotic decision support can be considered, structured offline evaluation is needed to assess whether model outputs align with auditable stewardship constraints under real-world admission contexts. We therefore evaluated whether post hoc LLM-generated empiric antibiotic recommendations showed greater concordance with a pre-specified stewardship benchmarking framework than clinician-initiated regimens in a retrospective shadow-mode setting. **Methods**: Single-center retrospective paired evaluation at Clinical Emergency Hospital of Bucharest (Internal Medicine, 2020–2024). The unit of analysis was the admission (N = 493), with paired 24 h empiric regimens (clinician-prescribed vs. post hoc LLM-recommended via OpenAI API; not visible to clinicians; no influence on care). Local laboratory-derived epidemiology was precomputed from microbiology exports and provided as structured prompt context to approximate information parity with clinicians’ implicit local ecology knowledge. Primary (prespecified) endpoint: any contextual guardrail violation (unjustified carbapenem/antipseudomonal/anti-MRSA under prespecified structured severity/MDR-risk rules), exact McNemar. Key secondary (prespecified): Δ contextual guardrail penalty (LLM − Clin), sign test and Wilcoxon signed-rank (ties reported). Ethics committee approval was obtained. **Results**: Guardrail violations occurred in 17.0% of clinician regimens vs. 4.9% of LLM regimens (paired RD −12.2%; matched OR 0.216, 95% CI 0.127–0.367; McNemar exact *p* = 1.60 × 10^−10^). Δ penalty had median 0 with 398/493 ties; among non-ties, improvements (Δ < 0) exceeded adverse shifts (79 vs. 16; sign-test *p* = 3.47 × 10^−11^). **Conclusions**: In this offline, non-interventional paired evaluation, LLM-generated empiric regimens showed greater concordance with a pre-specified stewardship benchmarking framework than clinician empiric regimens for the same admissions. These findings should not be interpreted as evidence of clinical superiority, patient safety, or causal effectiveness, but rather as process-level benchmarking within a rule-based stewardship construct. As such, reproducible guardrail-based benchmarking may serve as an early pre-implementation step to identify alignment and potential failure modes before prospective, safety-governed evaluation.

## 1. Introduction

Antimicrobial resistance (AMR) remains one of the most urgent global public health threats: bacterial AMR was estimated to be directly responsible for ~1.27 million deaths in 2019 and associated with ~4.95 million deaths worldwide in the same year [1]. Looking forward, the UK government–commissioned research projected up to 10 million deaths annually by 2050 in a worst-case scenario without effective action [2], while newer global forecasting work estimates around 1.91 million deaths attributable to AMR (and 8.22 million associated deaths) in 2050, underscoring a sustained and aging-driven burden trajectory [3]. This escalating crisis is a core rationale for antimicrobial stewardship programs, which aim to optimize antimicrobial use, particularly by limiting unnecessary broad-spectrum empiric exposure, while preserving timely, appropriate coverage when severe infection is suspected. Antimicrobial resistance can also be understood as an evolutionary response to antibiotic selection pressure, whereby microorganisms adapt through genetic variation and horizontal gene transfer to survive antimicrobial exposure, thus facing an informational challenge in adaptive survival under antimicrobial pressure [4,5]. In parallel, modern inpatient care confronts a complementary yet unresolved informational challenge: integrating rapidly expanding and heterogeneous clinical data, such as patient history, comorbidities, severity indicators, prior antibiotic exposure, and local epidemiology, into timely and precise empiric antimicrobial decision-making at the bedside [6,7]. Within this framing, large language models (LLMs) and other artificial intelligence (AI)-enabled decision-support approaches can be viewed as attempts to improve clinical information synthesis and decision consistency, while also introducing well-recognized safety risks such as hallucinated or unsupported recommendations that necessitate rigorous offline evaluation prior to any clinical deployment [8,9]. In this study, LLM recommendations were generated post hoc (offline), were not visible to clinicians, and had no influence on patient care. Whether such tools can support more context-sensitive empiric decision-making remains an open question that requires structured offline evaluation before any clinical deployment can be considered [10,11,12].

Because empiric prescribing decisions are often made under uncertainty (incomplete microbiological data at presentation, variable patient trajectories, and evolving local resistance patterns), stewardship evaluation benefits from focusing on a small set of “high-leverage” choices that disproportionately shape ecological impact and downstream resistance selection. In routine acute-care practice, three such decisions are the inclusion of (I) carbapenems, (II) antipseudomonal broad-spectrum β-lactams, and (III) anti-MRSA (Methicillin-resistant *Staphylococcus aureus*) agents. Many agents in these empiric categories fall into higher-stewardship priority groups (e.g., World Health Organization AWaRe “Access/Watch/Reserve” classes) and are consistently targeted by antimicrobial stewardship programs for indication review, risk-stratified initiation, and early de-escalation when structured risk factors are absent [13,14,15]. Carbapenems, in particular, are central to carbapenem-sparing strategies intended to reduce selective pressure for carbapenem resistance [16]. Anti-MRSA therapy is another common empiric add-on in hospitalized patients, yet multiple stewardship approaches (including rapid diagnostic–supported pathways) emphasize discontinuation when MRSA risk is low, to minimize unnecessary exposure and adverse effects, though a positive nasal swab may reflect colonization rather than active infection and its negative predictive value supports de-escalation in low-risk patients [17]. Finally, antipseudomonal therapy is widely recognized as a major driver of broad-spectrum pressure; stewardship guidance repeatedly stresses reserving such coverage for patients with convincing severity or MDR-risk signals and revisiting empiric breadth once early clinical and microbiological data accrue [13,18,19]. In this study, we therefore operationalized these high-impact stewardship choices as contextual guardrails, pre-specified “safety rails” that label broad-spectrum elements as justified only when objective, structured indicators of severity and MDR/MRSA risk are present.

Empiric antibiotic selection is not solely syndrome-driven but also contingent on local resistance ecology; therefore, facility-specific antibiograms and their syndrome-oriented extensions are routinely used to operationalize “what is likely to work here and now” [20]. In particular, the weighted-incidence syndromic combination antibiogram (WISCA) extends conventional antibiograms by integrating syndrome-specific pathogen incidence with susceptibility data to estimate the expected coverage of candidate empiric regimens (including combinations), and has been proposed as a pragmatic decision aid for empiric therapy [21,22,23].

Despite growing interest in LLMs for clinical decision support, evaluation frameworks for early-stage clinical AI emphasize transparent reporting, safety assessment, and non-interventional study designs prior to any real-world deployment [24,25]. This is especially salient in antimicrobial stewardship, where outputs may be clinically plausible yet unsafe or unjustified in specific contexts, and where empiric broad-spectrum escalation decisions are high-impact and heterogeneous under uncertainty [8,9,12].

LLM outputs can be sensitive to prompt context and may exhibit non-determinism and confidently stated but unsupported content (“hallucinations”), raising reproducibility and safety concerns in high-stakes prescribing decisions [8,9,26]. Reporting guidance for LLM-based clinical studies further reinforces the need for reproducible pipelines, clear definition of the evaluation setting, and explicit separation between model development and outcome assessment [24,25].

We therefore conducted a single-center, retrospective offline paired evaluation comparing clinician-prescribed empiric antibiotic regimens with post hoc LLM recommendations within the first 24 h of admission, using pre-specified structured contextual stewardship endpoints. Local laboratory-derived epidemiology was incorporated as a static information-parity component, acknowledging that clinicians acquire implicit institutional ecology knowledge through routine practice, whereas a general-purpose LLM does not.

Our objective was not to test clinical effectiveness or safety in patient outcomes, but to assess whether LLM-generated empiric recommendations would show greater concordance with a pre-specified stewardship benchmarking framework than clinician-initiated regimens for the same admissions. Because any future clinical deployment of LLM-guided empiric prescribing would require substantially stronger evidence, we position this study as an early pre-implementation benchmarking step: a non-interventional shadow-mode phase intended to assess stewardship alignment, reveal potential failure modes, and inform whether prospective, safety-governed evaluation is justified.

Accordingly, the primary (prespecified) outcome was any contextual guardrail violation (paired binary), and the key secondary (prespecified) outcome was the paired difference in contextual guardrail penalty (LLM − Clin). All additional analyses were considered secondary, supplementary, or exploratory and were interpreted with explicit multiplicity and selection-bias caution, without inference about patient-centered clinical benefit.

## 2. Results

### 2.1. Cohort Flow

**Figure 1:**
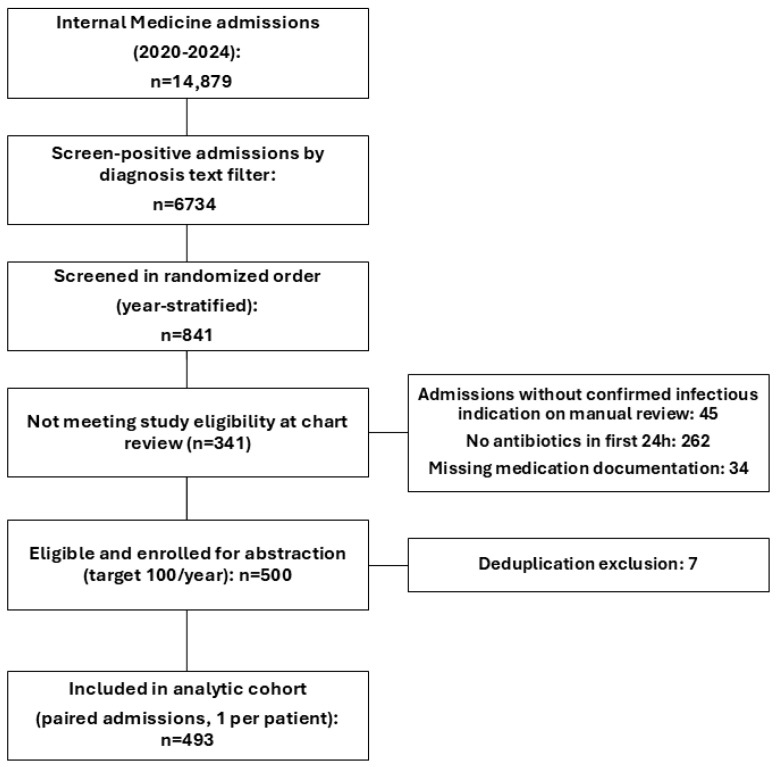
STROBE flow diagram of cohort selection (admissions as the unit of analysis). Numbers refer to admissions. Of 841 chart-reviewed admissions, 341 did not meet eligibility at chart review (admissions without confirmed infectious indication on manual review N = 45; no systemic antibiotics within 24 h N = 262; missing medication documentation N = 34). Of 500 enrolled admissions, 7 were removed by the prespecified deduplication rule (first eligible admission per patient), yielding a final paired analytic cohort of N = 493 admissions.

Among 14,879 Internal Medicine admissions at the Clinical Emergency Hospital of Bucharest (2020–2024), 6734 were identified using an infection-suspected admission diagnosis filter. To ensure feasibility of manual chart abstraction while maintaining balanced temporal coverage, we prespecified a target sample of 100 eligible admissions per year (total N = 500) as a pragmatic design choice. A total of 841 candidate admissions underwent manual chart review; during this process, prespecified exclusion criteria were operationalized at the record level (e.g., non-infectious admission, no systemic antibiotics within 24 h, missing medication documentation), with decision counts summarized in Supplementary Table S1. Of these, 500 admissions (100/year) met eligibility and were enrolled for detailed data abstraction. After applying the prespecified deduplication rule (first eligible admission per patient), 7 admissions were removed, yielding the final analytic cohort of 493 paired admissions. The remaining 341 screened admissions were not enrolled due to prespecified reasons (non-infectious admission N = 45; no systemic antibiotics within 24 h N = 262; missing medication documentation N = 34). See Figure 1.

Baseline cohort characteristics are summarized in Table 1. The median age was 72 years (IQR 64–82), 250/493 (50.7%) were female, and the median length of stay was 9 days (IQR 5–15). Community-onset infection predominated (459/493, 93.1%), and community-acquired pneumonia was the most common index syndrome (311/493, 63.1%). Overall, Table 1 shows that the paired cohort was predominantly older, community-onset, and pneumonia-weighted, with substantial early severity burden and relatively limited structured MDR-risk exposure. Endpoint mapping quality control identified no unmapped antibiotic agents in the analytic cohort.

The final paired analytic cohort included 493 admissions contributing matched clinician and LLM empiric regimens within the first 24 h. The predefined 72 h continued-therapy subset included 323 admissions, and the microbiology-evaluable paired subset included 158 admissions (Table 2).

### 2.2. Primary Endpoint

**Figure 2:**
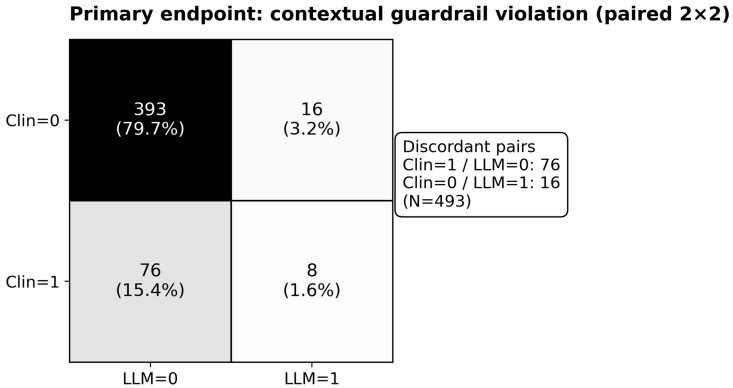
Primary endpoint (paired 2 × 2). Paired admission-level comparison (N = 493) of any contextual guardrail violation within the first 24 h: clinician regimen (rows) vs. LLM regimen (columns). Cells show counts and percentage of total admissions (N = 493). Effect estimates and *p*-values are reported in Table 2. Abbreviations: LLM, large language model; Clin, clinician.

In the paired cohort (N = 493), the primary prespecified endpoint: any contextual guardrail violation, occurred in 17.0% of clinician regimens versus 4.9% of LLM-recommended regimens (definitions, antibiotic mappings and weights in Supplementary Table S3). Discordant pairs favored the LLM arm (Clin = 1/LLM = 0: 76 vs. Clin = 0/LLM = 1: 16; 2 × 2: n00 = 393, n01 = 16, n10 = 76, n11 = 8), corresponding to a matched OR 0.216 (95% CI 0.127–0.367) and exact McNemar *p* = 1.60 × 10^−10^ (Table 2, panel A, Figure 2). These analyses were performed post hoc/offline; LLM recommendations were not visible to clinicians and did not influence care.

### 2.3. Key Secondary Endpoint

**Figure 3:**
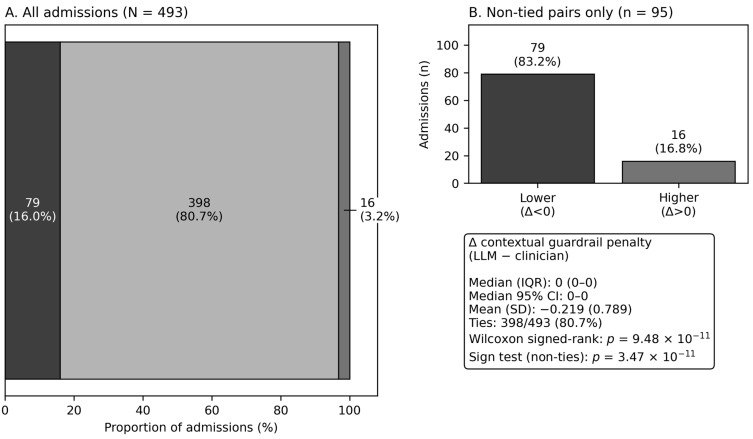
Key secondary prespecified endpoint: distribution of paired Δ contextual guardrail penalty (LLM − clinician) across admissions (N = 493). (**A**) All admissions, showing the proportion with lower (Δ < 0), tied (Δ = 0), and higher (Δ > 0) penalty in the LLM arm. (**B**) Non-tied pairs only (N = 95), highlighting the direction of discordance. Summary statistics report median (IQR), mean (SD), the number of ties, and paired tests (Wilcoxon signed-rank and sign test). Negative Δ indicates a lower contextual guardrail penalty in the LLM arm. Abbreviations: LLM, large language model; IQR, interquartile range; SD, standard deviation.

For the key secondary prespecified endpoint, the paired Δ contextual guardrail penalty (LLM − Clin) had a median of 0 (IQR 0–0) and mean −0.219 (SD 0.789). The penalty was lower in the LLM arm in 79 admissions, higher in 16, and tied in 398 (non-ties: 95). The paired distribution differed from zero by Wilcoxon signed-rank *p* = 9.48 × 10^−11^ and sign test *p* = 3.47 × 10^−11^ (Table 2, Panel B, Figure 3).

### 2.4. Secondary Endpoints (Multiplicity Caution)

These secondary analyses were prespecified as supportive rather than confirmatory: they help triangulate whether the primary stewardship signal is directionally consistent across related process measures, but because multiple endpoints were examined, they should not be interpreted as carrying the same inferential weight as the primary and key secondary outcomes.

#### 2.4.1. Contextual Guardrail Components

**Figure 4:**
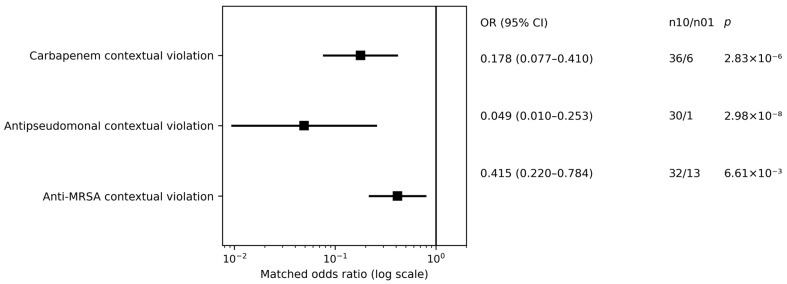
Secondary contextual guardrail components: matched odds ratios (paired analyses; N = 493). Squares indicate matched OR estimates and horizontal bars indicate 95% confidence intervals. Matched ORs are computed from discordant pairs (n10 = clinician = 1/LLM = 0; n01 = clinician = 0/LLM = 1) with 0.5 continuity correction and Wald 95% confidence intervals on the log scale. p-values are from the exact McNemar test. Values < 1 indicate fewer violations in the LLM arm. Secondary endpoints should be interpreted with multiplicity caution. Abbreviations: LLM, large language model; OR, odds ratio; CI, confidence interval.

In secondary component analyses (paired; multiplicity caution), discordant patterns generally favored fewer contextual violations in the LLM arm. Carbapenem contextual violations had 36 clinician-only vs. 6 LLM-only discordant pairs (matched OR 0.178, 95% CI 0.077–0.410; exact McNemar *p* = 2.83 × 10^−6^). Antipseudomonal contextual violations had 30 clinician-only vs. 1 LLM-only discordant pairs (matched OR 0.049, 95% CI 0.010–0.253; exact McNemar *p* = 2.98 × 10^−8^). Anti-MRSA contextual violations had 32 clinician-only vs. 13 LLM-only discordant pairs (matched OR 0.415, 95% CI 0.220–0.784; exact McNemar *p* = 6.61 × 10^−3^) (Table 2, Panel C; Figure 4).

Consistent with these component-level findings, overall broad-spectrum class exposure, defined as use of at least one carbapenem, antipseudomonal agent, or anti-MRSA agent within the first 24 h, was lower in the LLM arm. In the paired cohort (N = 493), any broad-spectrum class exposure occurred in 58.6% of clinician regimens versus 39.6% of LLM regimens, supporting the interpretation that LLM recommendations were, under this prespecified rule-based benchmarking framework, less likely to trigger broad-spectrum stewardship guardrails during early empiric management (Table 2, Panel C).

#### 2.4.2. Costs (Secondary)

Total empiric antibiotic costs over the first 24 h were lower in the LLM arm when aggregated across the cohort. Summing admission-level empiric antibiotic costs yielded 4689.02 EUR for clinician regimens versus 2591.83 EUR for LLM regimens (EUR, using the study’s fixed price mapping and exchange-rate assumptions). These totals are presented as descriptive cohort-level context and should be interpreted alongside the paired distributional analyses reported below. For more details, see Supplementary Data File S2 (data_file_s2_costing).

**Figure 5:**
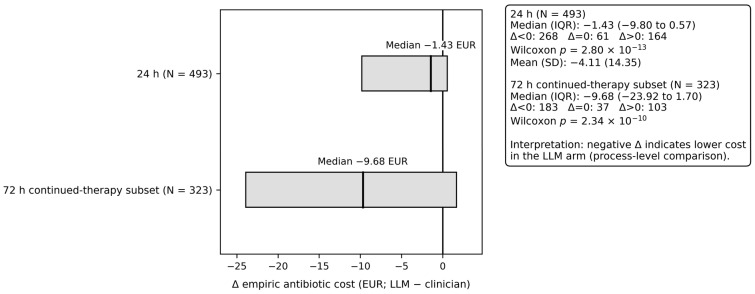
Secondary endpoint: paired empiric antibiotic cost deltas (Δ cost, EUR; LLM − clinician). The 24 h analysis includes all paired admissions (N = 493). The 72 h analysis is restricted to a prespecified continued-therapy subset in which empiric therapy initiated by the clinician was continued for ≥72 h (N = 323). Boxes indicate the interquartile range with the median; the vertical reference line marks Δ = 0. Negative Δ indicates lower empiric antibiotic cost in the LLM arm (process-level comparison). Abbreviations: LLM, large language model; EUR, euros; IQR, interquartile range.

At the admission level, the 24 h empiric antibiotic cost delta (LLM − clinician) had a median of −1.43 EUR (IQR −9.80 EUR to 0.57 EUR) (paired Wilcoxon *p* = 2.80 × 10^−13^; N = 493; sign test *p* = 6.41 × 10^−7^; N = 493). In the predefined 72 h continued-therapy subset (N = 323), the 72 h cost delta had a median of −9.68 EUR (IQR −23.92 EUR to 1.70 EUR) (paired Wilcoxon *p* = 2.34 × 10^−10^). These are process-level economic comparisons of recommended versus prescribed empiric regimens under the study’s costing assumptions and do not imply clinical outcome impact (Figure 5).

#### 2.4.3. Microbiology-Evaluable Subset

**Figure 6:**
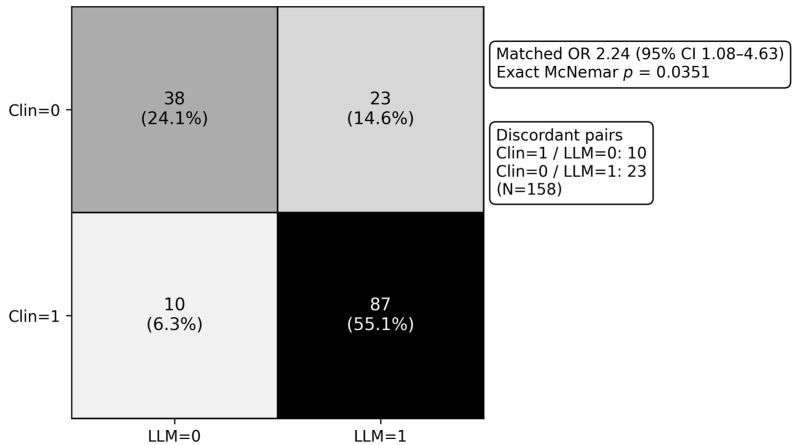
Microbiology-evaluable subset: paired active coverage against the index organism (N = 158). 0 = inactive coverage; 1 = active coverage against the index organism (per prespecified definition). Each cell shows the count (and percentage) of admissions classified as inactive/active for the clinician regimen (rows) versus the LLM regimen (columns), under the study’s prespecified definition of “active coverage” against the index organism. Discordant pairs (clinician active/LLM inactive vs. clinician inactive/LLM active) are shown with the matched OR and exact McNemar p-value. Because evaluability depends on culture availability and interpretable susceptibility, this analysis reflects subset-level process benchmarking rather than cohort-wide effectiveness. Abbreviations: LLM, large language model; OR, odds ratio; CI, confidence interval.

In the microbiology-evaluable paired subset (N = 158, with 335/493 not eligible for microbiological evaluation), active coverage against the index organism differed between arms (2 × 2: n00 = 38, n01 = 23, n10 = 10, n11 = 87), with a matched OR 2.24 (95% CI 1.08–4.63) and exact McNemar *p* = 0.0351. Culture acquisition and positivity are post-baseline processes and may correlate with severity/trajectory; therefore, this subset analysis does not estimate causal effectiveness. Because evaluability depends on culture availability and interpretable susceptibility, these findings should be interpreted as subset-level process benchmarking rather than cohort-wide effectiveness (Figure 6).

#### 2.4.4. Concordance (Supplementary Framing)

Regimen concordance between clinicians and the LLM was limited: 57/493 (11.6%) were identical as an antibiotic set, 137/493 (27.8%) shared the same primary agent, 148/493 (30.0%) had any antibiotic overlap, and 345/493 (70.0%) had no overlap (see Supplementary Table S13).

#### 2.4.5. Supplementary QC Note (NO_ANTIBIOTIC)

The LLM recommended NO_ANTIBIOTIC in 5/493 admissions. None activated the structured SEVERE or MDR_RISK trigger pattern used in the contextual guardrail framework. Case-level details are provided in Supplementary Table S4. Per study definition, all five admissions had received empiric antibiotics from the clinician, whereas the LLM recommended withholding antibacterial therapy.

#### 2.4.6. Exploratory Modeling (Supplement Only)

Exploratory complete-case models (N = 493) were used to assess predictors of a nonzero Δ contextual guardrail penalty (LLM − Clin ≠ 0; events = 95) using logistic regression with robust (HC3) standard errors. Given the retrospective observational design, potential sparsity in some covariate levels, and the exploratory intent, these analyses are presented in the Supplementary Materials only and interpreted cautiously (Table S5a; model-level complete-case QC in Table S6a).

## 3. Discussion

### 3.1. Principal Findings

In this single-center retrospective offline/post hoc paired evaluation of 493 admissions, LLM-generated empiric regimens produced fewer contextual guardrail violations than clinician regimens within the first 24 h (4.9% vs. 17.0%, paired RD −12.2%; matched OR 0.216; exact McNemar *p* = 1.60 × 10^−10^). The reduction was directionally consistent across guardrail components (antipseudomonal, carbapenem, anti-MRSA), and coincided with lower broad-spectrum class use (58.6% vs. 39.6%) and a modest reduction in 24 h empiric antibiotic costs (median Δ −1.43 EUR, IQR −9.80 EUR to 0.57 EUR; bootstrap 95% CI −4.09 EUR to −0.07 EUR), while overall early exposure was similar (median Δ DDD/24 h 0 with substantial ties). These patterns are directionally consistent with core antimicrobial stewardship objectives, but they remain process-level signals and should not be interpreted as direct evidence of superior patient outcomes [27,28,29].

From a stewardship perspective, the signal evoked from the data is clinically plausible: unnecessary broad-spectrum exposure is a well-established driver of avoidable harms (notably *C. difficile* risk) and selection pressure for resistant organisms, and stewardship interventions have been associated with reductions in both CDI and resistant organism infection/colonization in hospital settings [27,29]. Broad-spectrum “intensity” measures have also shown dose–response relationships with hospital-associated CDI risk, reinforcing the biological rationale for prioritizing spectrum-aware empiric choices when patient risk factors do not mandate escalation [30].

### 3.2. Interpretation in Antimicrobial Stewardship Terms

The central interpretive point is that this study evaluates concordance with a pre-specified stewardship benchmarking framework, not clinical superiority of LLM recommendations over clinician care. In this study, the primary endpoint operationalizes contextual appropriateness constraints (“local stewardship guardrails”), i.e., whether empiric regimens cross prespecified escalation thresholds (antipseudomonal, carbapenem, anti-MRSA) in the absence of documented high-risk features, thereby capturing a proximal marker of prescribing quality rather than downstream outcomes. Accordingly, the observed advantage in the LLM arm is best interpreted as greater alignment with the benchmark itself rather than proof of better clinical care. This distinction is essential because process-level stewardship alignment, while relevant to stewardship objectives, is not interchangeable with patient-centered effectiveness or safety. Consistent with this framing, the LLM arm showed fewer contextual guardrail violations (4.9% vs. 17.0%), fewer discordant escalations, and lower broad-spectrum class use (58.6% vs. 39.6%), alongside a modest reduction in 24 h costs (median Δ −1.43 EUR), while overall early exposure (DDD/24 h) remained centered at 0—suggesting that the main effect is spectrum selection rather than “less antibiotic” per se [15].

Nuancing the comparison is essential: the clinician arm reflects real-world empiric prescribing under diagnostic uncertainty, evolving information, and local organizational constraints, where clinicians may rationally “over-cover” to avoid the well-described harms of inappropriate empiric therapy in severe bacterial infections. Meta-analyses across serious infections (e.g., pneumonia/BSI/sepsis) have repeatedly associated inappropriate initial empiric antibiotics with higher mortality, which provides a plausible clinical rationale for risk-averse escalation when pathogen and susceptibility data are unavailable [31,32]. Conversely, stewardship frameworks (including WHO’s AWaRe system) explicitly emphasize minimizing unnecessary Watch/Reserve exposure to reduce resistance selection pressure, providing a principled basis for guardrails that discourage broad-spectrum escalation when not clearly indicated [15]. Taken together, these endpoints and findings align with the study’s central stewardship trade-off: maximizing the probability of appropriate early coverage while minimizing avoidable broad-spectrum exposure, but they do so as an auditable prescribing-process benchmark rather than an outcomes trial.

### 3.3. Why Might the LLM Look Better on Guardrails?

One plausible explanation is framework dependence. The LLM is being evaluated against a rule-defined stewardship construct, and LLM outputs may preferentially reproduce guideline-like defaults when high-risk features are not explicit in the structured context. Under those conditions, a model prompted with a standardized admission summary may preferentially select narrower empiric options unless it encounters clear triggers for escalation, thereby increasing concordance with prespecified guardrails. This interpretation is also consistent with prior work on antimicrobial clinical decision support systems, where the most reproducible effects have generally been observed on process-level endpoints such as prescribing appropriateness or reduced unnecessary broad-spectrum exposure, rather than on hard clinical outcomes [20,33,34].

The pattern observed in this study is consistent with that interpretation. For the primary prespecified endpoint, the observed difference was driven mainly by asymmetric discordant pairs, with substantially more admissions in the “Clinician = 1/LLM = 0” cell than in the reverse direction (76 vs. 16). A similar pattern was seen for the key secondary endpoint, where lower contextual guardrail penalty in the LLM arm was more common than higher penalty (79 vs. 16 among non-tied pairs, with 398 ties overall). This asymmetry supports the interpretation that, under the structured admission context used here, the LLM more often defaulted toward less escalated empiric regimens. However, the 16 admissions moving in the opposite direction remain clinically important: they are precisely why a favorable benchmark signal cannot be equated with autonomy, safety, or clinical superiority.

An additional caution concerns the small subset of admissions in which the LLM recommended no antibiotic therapy at the initial empiric decision point. In this dataset, five such NO_ANTIBIOTIC cases were identified and are summarized in Supplementary Table S4. Although these cases did not meet the structured early severity or MDR-risk trigger logic used in the benchmarking framework, this should not be interpreted as evidence that withholding empiric therapy would have been clinically safe. Rather, these admissions illustrate a key boundary of the present study design: a favorable result within a rule-based stewardship benchmark does not resolve the bedside trade-off between avoiding unnecessary exposure and preventing under-treatment in ambiguous early presentations.

By contrast, the clinician arm reflects real-world empiric prescribing under time pressure, incomplete information, and institutional constraints, where the perceived downside of under-treatment may rationally dominate. In settings where sepsis-related concerns and escalation norms are salient, broader empiricism may reflect precaution under uncertainty rather than poor practice [35]. More broadly, prior work in antibiotic decision-making has described how uncertainty, anticipated regret, and action bias can tilt prescribing toward “just-in-case” treatment, which is directionally consistent with why more clinician regimens may cross broad-spectrum guardrails in routine care [36,37]. The present findings therefore narrow the claim rather than eliminate its value: this study provides an auditable benchmark of stewardship alignment in a shadow-mode setting, not proof that LLM-guided care would be clinically better if implemented.

### 3.4. Microbiology-Evaluable Subset: What It Means and What It Does Not Mean

In the microbiology-evaluable paired subset (N = 158, with 335/493 not eligible for microbiological evaluation), empiric active coverage against the index organism differed between arms (2 × 2: n00 = 38, n01 = 23, n10 = 10, n11 = 87), corresponding to a matched OR 2.24 (95% CI 1.08–4.63) with exact McNemar *p* = 0.0351. These data suggest that, among admissions where an organism and interpretable susceptibility were available, the LLM-generated regimen more often met in vitro activity criteria than the clinician regimen within the empiric window.

However, microbiology “evaluability” is not a baseline attribute of the cohort; it depends on culture acquisition, organism recovery, and interpretable susceptibility, processes that occur during care and are influenced by clinical severity, diagnostic strategy, prior antibiotic exposure, and workflow factors. Conditioning inference on such a post-baseline filter can induce selection (collider-stratification) bias, meaning that associations within the evaluable subset may not reflect cohort-wide performance and should not be interpreted as causal evidence of superior effectiveness [38,39]. In this context, the most defensible interpretation is that the microbiology subset provides subset-level process benchmarking (i.e., an auditable “coverage check” when microbiology is available) and is best viewed as hypothesis-generating, rather than as a definitive estimate of empiric effectiveness across all admissions.

To strengthen transparency, we treated microbiology evaluability as a selection process. We therefore (i) compared baseline characteristics and severity proxies between microbiology-evaluable and non-evaluable admissions (Table S2), and (ii) interpreted microbiology-subset findings as supportive evidence of prescribing-process alignment rather than cohort-wide effectiveness. Future prospective shadow-mode (offline, non-interventional) validation could predefine culture-based endpoints and consider methods that address evaluability mechanisms (e.g., inverse-probability weighting), while prioritizing patient-centered outcomes.

### 3.5. Economic and Exposure Endpoints (Cost and DDD)

Although the median per-admission 24 h empiric antibiotic cost difference was modest (LLM − Clin −1.43 EUR), cohort-level totals illustrate the potential budget impact of spectrum selection. Across all 493 admissions, 24 h anti-infective acquisition costs were EUR 4689.02 in the clinician arm versus EUR 2591.83 in the LLM arm (absolute difference EUR 2097.19, −44.7%), corresponding to a mean reduction of EUR 4.25 per admission. In the prespecified 72 h continued-therapy subset (N = 323), totals were EUR 8676.20 versus EUR 4636.98 (difference EUR 4039.22, −46.6%), corresponding to EUR 12.50 per admission in that subset; when scaled to the full cohort, this corresponds to EUR 8.19 per admission (N = 493). These figures reflect drug acquisition costs only (excluding administration, monitoring, and downstream consequences), yet they are directionally consistent with the broader health-economic literature indicating that stewardship interventions can reduce antibiotic expenditures and may avert costly complications linked to broad-spectrum exposure (including hospital-associated *Clostridioides difficile* infection) [29,40,41].

In contrast, DDD/24 h differences were small (median Δ 0, with substantial ties), suggesting that the stewardship signal is driven primarily by spectrum choice rather than a reduction in overall antibiotic intensity (Table S12). This interpretation aligns with WHO’s AWaRe framework, which encourages minimizing unnecessary Watch/Reserve exposure as a stewardship objective while maintaining adequate early therapy when clinically warranted.

### 3.6. Comparison with Prior Work

The recent literature on AI support for antibiotic decisions spans two partly separate traditions: (i) rule-based or ML-driven clinical decision support systems (CDSS) embedded in stewardship programs, and (ii) generative LLM evaluations that typically test narrative recommendations against guidelines or expert ratings. For CDSS, the best-supported effects tend to be on process outcomes (improved guideline concordance or reduced unnecessary broad-spectrum prescribing) while evidence for consistent patient-outcome benefits is mixed and highly context-dependent [34]. In parallel, LLM-focused work has expanded quickly but remains heterogeneous in design and endpoints, with recurring concerns about standardization, evaluation bias, and the gap between “plausible-sounding” recommendations and bedside usability [42]. Prior research positioned chatbots as potentially useful adjuncts to stewardship, while emphasizing the need for governance, validation, and human oversight, concerns that are directly addressed by the shadow-mode, offline nature of the current study [10,11,12].

Most empirical LLM studies in infection-related decision-making have relied on vignettes or guideline-question formats, reporting accuracy, completeness, or safety scoring rather than audited, admission-level paired comparisons. For example, LLMs have been assessed for antibiotic advice in general practice scenarios and for guideline compliance in pneumonia-related questions, with conclusions typically framed as “promising but not reliable without oversight” [43]. Beyond vignettes, a key step forward has been evaluation on real clinical notes: Williams et al. tested LLM recommendations on emergency department documentation for several clinical tasks, including antibiotic prescription status, highlighting both capability and the need for careful evaluation design [44]. Within stewardship-adjacent workflows, recent work has also explored more constrained tasks (e.g., antimicrobial classification at scale, or structured treatment recommendations anchored to rapid diagnostic outputs), where LLM performance can be strong but still depends on local framing and safeguards [45]. Collectively, this body of work supports a cautious interpretation: LLMs may reproduce guideline-like defaults and can assist with standardized outputs, yet they require domain constraints, quality control, and evaluation against clinically meaningful processes.

Against that backdrop, this study’s contribution is the shift from “Is the answer correct in a vignette?” to “How do LLM recommendations compare to the clinician’s regimen for the same admission, under a prespecified stewardship lens?” Compared with prior LLM evaluations that focus on vignette accuracy or unpaired comparisons, this work adds a paired admission-level offline-mode benchmark with locally defined guardrails and auditable reproducibility artifacts. The paired design (N = 493 admissions) reduces confounding by case-mix, while the endpoint definition: contextual guardrail violations and penalties, makes explicit the stewardship trade-off between adequate empiric coverage and avoiding unnecessary escalation. This also directly addresses a recurrent critique in the LLM-healthcare literature: that “accuracy” metrics are not interchangeable with operational safety, and that evaluation must be tied to the intended clinical process and context [42].

Two additional features further distinguish the approach implemented in this research. First, the study embeds decision-making in local constraints (formulary, local epidemiology priors/guardrails), which is often missing from generic LLM demonstrations and is a major determinant of stewardship appropriateness. Second, the analysis is unusually audit-ready: locked cohorts, prespecified endpoints, QC checks, and reproducible artifacts allow reviewers to interrogate robustness rather than infer it. That combination: paired shadow-mode evaluation, locally meaningful guardrails, and reproducibility engineering, puts the research closer to the methodological standard expected in higher-tier venues than many early LLM vignette papers, while still appropriately positioning the evidence as process benchmarking rather than outcomes validation [34].

### 3.7. Strengths

Several methodological strengths support the interpretability and reproducibility of this work. First, the paired admission-level design (clinician vs. LLM regimen for the same admission; N = 493) reduces confounding by case-mix and makes treatment contrasts less sensitive to secular changes in practice or patient mix across the 2020–2024 sampling frame. Second, the study uses pre-specified, operational guardrail endpoints that reflect core stewardship priorities: avoiding unnecessary escalation to antipseudomonal, carbapenem, and anti-MRSA therapy in the absence of documented high-risk features, thereby translating stewardship principles into auditable, admission-level metrics and aligning evaluation with the intended clinical process rather than with decontextualized “accuracy” alone [24,25,42]. Third, the analysis pipeline was built for auditability: a locked cohort, structured inputs/outputs, reproducible scripts, file hashing/manifesting, and explicit QC checks (including complete endpoint mapping QC in the final dataset) reduce the risk of undisclosed analytic flexibility and facilitate independent verification, which is particularly important in early-stage clinical AI evaluation and LLM reporting [24,25]. Finally, conclusions are supported by complementary endpoints that triangulate the same stewardship signal from different angles: binary violation rates, ordinal/penalty deltas with many ties, broad-spectrum class use composites and components, acquisition-cost deltas at 24 h (and a prespecified 72 h continued-therapy subset), and DDD/24 h, providing convergent evidence about spectrum selection behavior rather than reliance on a single metric, consistent with prior process-focused antimicrobial decision-support evaluation [34].

### 3.8. Limitations

The first interpretive limitation is framework dependence. The primary and key secondary endpoints were defined within a pre-specified rule-based stewardship construct, and LLM outputs may preferentially reproduce guideline-like defaults when escalation triggers are not explicit in the structured context. Under such conditions, the model may appear to “perform better” partly because it is being evaluated against criteria that are intrinsically closer to codified stewardship logic than to the full uncertainty of real-world bedside decision-making. We therefore interpret the observed advantage in the LLM arm as greater concordance with the benchmarking framework rather than independent evidence of clinical superiority, patient safety, or causal effectiveness. This limitation does not nullify the study’s value, but it narrows the claim: the present work is best understood as an auditable pre-implementation benchmark rather than an outcomes-based test of clinically better care.

Beyond this framework dependence, the study has additional limitations that should be considered when interpreting the findings. As an observational post hoc analysis, it does not establish clinical effectiveness or safety in patient outcomes, nor does it support causal claims about what would have happened had the LLM recommendations been implemented. The clinician regimen reflects real practice, whereas the LLM regimen is a counterfactual comparator generated offline; differences therefore quantify process concordance with prespecified stewardship guardrails, not treatment effects on morbidity or mortality [46].

Generalizability is constrained by context and study size. This was a single-center study from a Romanian tertiary emergency hospital, meaning absolute rates (e.g., violation frequencies, cost deltas) may depend heavily on local formulary constraints, unit prices, prescribing culture, stewardship norms, and resistance ecology (a locally derived epidemiology prior). Additionally, the analytic cohort was derived from an infection-suspected sampling frame and therefore does not represent all Internal Medicine admissions. The sample size was determined by the prespecified chart-review capacity and year-stratified sampling plan; precision for paired effect estimates is appropriately reflected in the reported confidence intervals. Accordingly, external validity is uncertain; multicenter replication and prospective shadow-mode evaluation are needed before drawing conclusions about broader applicability. That said, the evaluation framework: paired admission-level benchmarking using transparent guardrails, is portable, even if guardrail definitions and cost inputs require local re-specification.

Retrospective EHR data introduce measurement error and documentation bias. Manual abstraction was performed by a single investigator; while supervised and QC-checked, residual abstraction error and documentation bias may persist and could affect syndrome labels, severity proxies, and guardrail triggers. Syndrome labels, severity proxies, and “risk modifier” flags are susceptible to misclassification and incomplete documentation, which can influence guardrail adjudication. For structured variables defining guardrail triggers (e.g., severe sepsis criteria, comorbidities), the absence of documentation in the electronic health record was pragmatically treated as the absence of the condition, reflecting standard clinical prescribing constraints under uncertainty. In particular, guardrails that trigger escalation only when high-risk features are documented can create asymmetry: a clinician may have acted on bedside cues or evolving clinical trajectory that were not captured in the structured record, whereas the LLM is necessarily limited to recorded variables and cannot replicate physical examination, real-time reassessment, or dynamic response to therapy. This limitation cuts both ways: documentation gaps can make clinician choices appear “non-concordant” with guardrails, while also restricting the LLM from safely escalating when unrecorded risk is present.

Furthermore, because some severity/support markers may be recorded after initial antibiotic initiation within the 24 h window, information parity at the exact decision timestamp cannot be fully guaranteed. This potential “look-ahead” bias may skew estimates in favor of the LLM and reinforces that results quantify rule-based concordance within a 24 h management frame, not causal clinical effectiveness.

Multiplicity and endpoint hierarchy require explicit framing. Although the primary endpoint and key secondary endpoint were prespecified, additional secondary endpoints (components, composites, cost, DDD, subsets) increase the risk of false-positive findings if over-interpreted. We therefore state explicitly which endpoints are prespecified (primary + key secondary) versus supportive/exploratory, and for the prespecified paired-delta family we report Holm-adjusted *p*-values and interpret them cautiously [47]. In practical terms, the secondary endpoints are intended to show consistency of direction across related stewardship-process measures, not to function as a collection of independent confirmatory claims.

Subset analyses are particularly vulnerable to selection effects. The microbiology-evaluable subset (N = 158; 335/493 not eligible for microbiological evaluation) is defined by post-baseline processes, culture acquisition, organism recovery, and interpretable susceptibility, which correlate with severity, diagnostic workup, and prior antibiotics. Conditioning on evaluability can induce collider/selection bias, so differences in coverage within this subset should be interpreted as subset-level process benchmarking and hypothesis-generating, not cohort-wide effectiveness [39,48].

Economic estimates are partial and setting-dependent. The cost analysis reflects drug acquisition costs (based on local unit pricing assumptions) within 24 h (and a prespecified 72 h continued-therapy subset), and does not capture administration costs, monitoring, adverse events, downstream complications, or opportunity costs. Any extrapolation to institutional budgets should be presented as scenario analysis with explicit assumptions rather than as observed savings.

Finally, because LLM outputs are model/version and prompt-context dependent, performance may vary across systems, time, and governance constraints; any clinical translation would require prospective validation, human oversight, and safety monitoring under an approved protocol.

### 3.9. Implications and Next Steps

Placed on a translational evidence pathway, the present study corresponds to an early Phase 0 pre-implementation benchmark rather than a test of clinical effectiveness. Its value lies in showing that LLM-generated empiric regimens can be audited against explicit stewardship constraints in a shadow-mode setting without influencing patient care, thereby informing whether more rigorous prospective evaluation is warranted. This study is not designed to change clinical practice directly; rather, it provides a structured evidence base for deciding whether more clinically proximate evaluation is justified.

**Figure 7:**
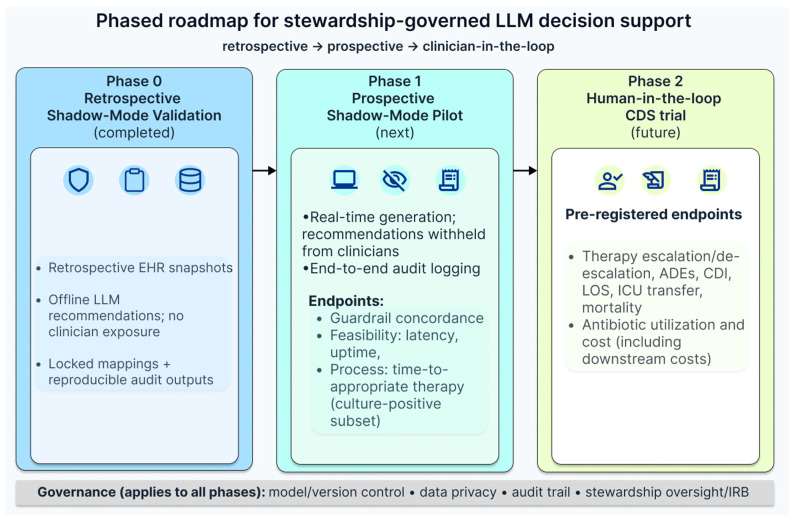
Phased roadmap for stewardship-governed translation of LLM-based empiric antibiotic decision support. Phase 0 corresponds to the present retrospective study, implementing admission-level shadow-mode benchmarking with locally defined guardrails and cost/DDD endpoints, without clinician exposure. Phase 1 illustrates a prospective shadow-mode pilot in which real-time LLM recommendations remain withheld from clinicians, while audit logging, drift monitoring, and prespecified process endpoints are evaluated. Phase 2 depicts a subsequent human-in-the-loop clinical decision support (CDS) trial with stewardship oversight and pre-registered outcome-focused endpoints. Blue, teal, and green panels denote the sequential translational phases, horizontal arrows indicate the proposed progression between phases, and the gray governance bar indicates requirements applying across all phases. The diagram is conceptual and does not imply that clinical outcome improvement was assessed in the retrospective phase. Abbreviations: LLM, large language model; CDS, clinical decision support; IRB, institutional review board; DDD, defined daily dose.

These findings support further prospective validation under governance safeguards, with outcome-focused endpoints and stewardship oversight. A pragmatic next step is a prospective shadow-mode pilot in which LLM recommendations are generated in real time but remain hidden from clinicians, allowing robust monitoring of model stability, data-flow integrity, and safety signals without influencing care. This phase can also predefine and validate operational endpoints (e.g., guardrail concordance, spectrum class use, time-to-appropriate therapy when microbiology is available) and confirm the feasibility of capturing key covariates reliably (severity proxies, source control, allergy history, renal dosing constraints) under routine workflow (Figure 7).

If shadow-mode performance remains acceptable, the subsequent step would be a human-in-the-loop stewardship deployment rather than autonomous decision-making: the model functions as a second opinion that presents one or more empiric options with explicit rationale and guardrail-aware warnings (e.g., “anti-MRSA not indicated without X/Y/Z”; “carbapenem reserved unless ESBL risk features present”), while the final decision remains with the clinician and/or stewardship team. This design aligns with established antimicrobial stewardship principles and helps mitigate known risks of LLMs (hallucination, overconfidence, context omission) through structured constraints, audit logging, and escalation pathways to infectious diseases or stewardship review when recommendations deviate from policy.

The present study evaluated only the initial 24 h empiric decision point and did not assess de-escalation timing or antibiotic modification after early clinical and microbiological data became available; prospective evaluation should therefore include time-to-de-escalation as a prespecified process endpoint.

Critically, prospective evaluation should be pre-registered with a clear endpoint hierarchy and analysis plan to minimize analytic flexibility and to support credible inference. Where feasible, a stepped-wedge or cluster-randomized design could compare clinician-only care versus CDS-supported care while tracking both process endpoints (appropriateness, broad-spectrum use, de-escalation timing) and patient-centered outcomes (clinical deterioration, ICU transfer, length of stay, adverse drug events, CDI, mortality), alongside economic endpoints that capture downstream costs rather than drug acquisition alone. Finally, governance should include explicit model version control, periodic recalibration against local epidemiology, and data protection measures to ensure that any translation to clinical practice remains safe, compliant, and clinically acceptable.

## 4. Materials and Methods

### 4.1. Study Design, Setting, and Timeframe

We conducted a single-center, retrospective, post hoc paired evaluation of empiric antibiotic regimens prescribed during the first 24 h of admission in an Internal Medicine department at Clinical Emergency Hospital of Bucharest (Spitalul Clinic de Urgență București; commonly known as “Floreasca”) across 2020–2024. Recommendations from a large language model were generated offline after care was delivered, were not visible to clinicians, and did not influence clinical management (non-interventional evaluation). Reporting followed the STROBE framework for observational studies [46].

Unit of analysis was the admission (crt_id: a de-identified study admission identifier), with paired regimens per admission: (i) the clinician-prescribed empiric regimen and (ii) the post hoc LLM-recommended regimen, each defined over the first 24 h. The paired within-admission design reduces confounding from time-invariant admission-level characteristics, since each admission serves as its own comparator.

### 4.2. Participants: Source Population, Sampling, and Flow

#### 4.2.1. Source Population Sampling Frame

The source population comprised 14,879 Internal Medicine admissions at Clinical Emergency Hospital of Bucharest between 2020 and 2024. Using an infection-suspected filter applied to admission diagnoses, 6734 candidate admissions were identified for potential eligibility screening. The infection-suspected screening frame was constructed using free-text admission diagnoses. An LLM-assisted categorization was used only as a clerical aid to pre-sort diagnosis strings into broad syndromic buckets (e.g., UTI, LRTI/pneumonia, IAI, SSTI, sepsis syndromes, COVID, other infections, non-infectious). Importantly, this automated pre-sorting was not used to determine eligibility. All chart-reviewed candidate admissions underwent manual verification of the admission documentation by the investigator, and final classification as infectious vs. non-infectious (and all inclusion/exclusion decisions) was made manually during chart review; therefore, no admission entered the analytic cohort based solely on an automated label. Any residual misclassification at the screening-frame stage would affect only which cases were prioritized for chart review, not the paired endpoint computation within the final manually verified analytic cohort.

#### 4.2.2. Random Sampling and Manual Screening (Year-Stratified)

Within each calendar-year stratum, candidate admissions were randomly ordered using a spreadsheet random number generator (Google Sheets RAND()) and then manually screened in that randomized order. The target sample size of 500 admissions (100 per year) was chosen pragmatically on the basis of manual chart-review capacity while preserving balanced year-stratified sampling across 2020–2024. No formal a priori power calculation was used for cohort construction; statistical precision is therefore conveyed by the reported confidence intervals around the paired effect estimates. The randomized ordering was retained as a locked snapshot (exported and archived) before detailed chart abstraction to preserve auditability of the screening sequence. A total of 841 candidate admissions underwent manual chart review. Of these, 500 were enrolled (100 per year) for detailed data abstraction. After applying a prespecified deduplication rule (one admission per patient within the sampled frame, defined as the first eligible admission), the final analytic cohort comprised 493 paired admissions.

#### 4.2.3. Eligibility Assessment and Reasons for Non-Inclusion

During chart-review screening, non-enrollment was categorized using prespecified chart-based reasons: non-infectious admission; no systemic antibiotics within 24 h; and missing medication documentation (reported in the STROBE flow diagram and Supplementary Table S1). Separately, after enrollment of 500 eligible admissions (100/year), a prespecified deduplication step (one admission per patient within the sampled frame, defined as the first eligible admission) removed 7 admissions, yielding the final analytic cohort of 493 paired admissions.

A de-identified screening log (screen_id, year, randomized order value, inclusion decision, and categorized non-inclusion reason) is provided as Supplementary Data Text S1.

#### 4.2.4. Bias and Representativeness

To reduce selection bias and ensure temporal coverage, screening was performed in a year-stratified random order with a fixed annual quota (100 admissions/year). The study was retrospective and single-center, and the analytic cohort represents infection-suspected admissions meeting eligibility criteria and requiring empiric antibiotics within 24 h; findings are therefore intended as process-level benchmarking and may not generalize to other institutions with different case-mix or resistance ecology.

### 4.3. Data Sources and Manual EHR Abstraction

#### Data Sources and Measurement

Clinical variables were abstracted from the hospital electronic health record (Hipocrate, Romania) and associated laboratory systems into a structured analytic dataset (data.xlsx) using a pre-specified data dictionary with structured fields whenever feasible. Abstraction captured (as available) demographics, comorbidities, severity proxies within 24 h, healthcare exposure/MDR-risk variables, prior microbiology flags, and antibiotic agents initiated within the first 24 h.

No formal inter-rater reliability exercise was prospectively documented; however, abstraction decisions were informally supervised by senior clinicians and were complemented by rule-based consistency checks within the analysis pipeline. To mitigate single-abstractor bias, we used a prespecified data dictionary, locked variable definitions prior to extraction, and implemented automated QC flags (range checks, completeness checks, and antibiotic-code mapping audits) that were reviewed prior to endpoint computation. Quality control (QC) procedures included (1) completeness checks for all variables contributing to primary and secondary endpoints; (2) range and plausibility checks for numeric fields (e.g., dose, duration); (3) automated detection of unmapped or deprecated antibiotic codes using a locked reference dictionary; and (4) generation of QC flags that informed predefined exclusion criteria for stewardship-related analyses.

Missingness for all key structured variables (baseline covariates and all fields required for endpoint computation) is summarized in Supplementary Table S6 (n missing and % missing per variable). Model-level missingness and complete-case QC for the exploratory regression analyses are provided in Supplementary Table S6a. Primary and key secondary endpoints were computed without imputation; analyses were performed as complete cases with respect to the variables required for each endpoint module, and denominators are reported per analysis.

### 4.4. Local Epidemiology Module (Laboratory Exports → Structured Prompt Context)

Because empiric antibiotic selection is influenced by facility-specific resistance ecology, we constructed a local epidemiology context module from hospital microbiology and susceptibility exports (laboratory information system). This module summarizes local pathogen/resistance patterns relevant to empiric therapy and is conceptually aligned with syndrome-oriented extensions of antibiograms, including weighted-incidence syndromic combination antibiograms (WISCA), which integrate syndrome-specific organism incidence with susceptibility to estimate expected coverage of candidate regimens (including combinations) [49].

To support information parity, the epidemiology module was injected into the LLM prompt as a static structured context block, reflecting the pragmatic notion that clinicians acquire institution-specific ecology knowledge through formal antibiograms, stewardship feedback loops, and routine practice, whereas a general-purpose LLM does not. Specifically, the module was precomputed from hospital laboratory exports and supplied to the model as a ward-year structured JSON/text block summarizing local pathogen frequencies and regimen-level susceptibility/coverage estimates relevant to empiric therapy selection. The intent was to provide the LLM with codified local ecology context, not patient-specific microbiology unavailable at the time of empiric prescribing. This strategy reduces one form of information asymmetry, but remains an approximation: clinicians may also rely on tacit knowledge, bedside cues, and informal institutional memory not captured in the structured block, whereas the model only receives the codified summary. Accordingly, the epidemiology module should be interpreted as a pragmatic local-ecology proxy for benchmarking purposes rather than a real-time or fully symmetric representation of all information available to clinicians.

Because the local epidemiology module was derived from annual ward-level laboratory exports, admissions occurring early in a given calendar year may have been exposed to contextual data reflecting isolates identified later in that same year. The module was used exclusively as static supportive context to approximate institutional ecology awareness and was not used to define endpoints or tuned to study metrics.

Considering that local resistance ecology can legitimately justify broader empiric therapeutic strategies than guideline-derived defaults in certain institutional environments, the integration of a local epidemiology module was methodologically required to ensure that the LLM was not evaluated under artificially ecology-blinded conditions.

### 4.5. LLM Recommendation Generation (OpenAI API) and Reproducibility/Auditability

Time zero was defined as hospital admission. The clinician regimen was defined as the set of systemic antibiotics initiated within the first 24 h after admission (the 0–24 h empiric management window). Clinician empiric regimens were derived from the pharmacy dispensing request records for the initial empiric order set. To ensure comparability with the single-time-point LLM recommendation, we verified that agents included in a multi-drug regimen were initiated concurrently (i.e., no sequential add-ons occurred within the 0–24 h window in the curated cohort). To mirror this definition, the LLM prompt was constructed from de-identified structured variables documented during the same 0–24 h window (e.g., admission labs and prespecified severity/support flags), and the model was asked to recommend an empiric regimen for that window under a shadow-mode, non-interventional design. Because both arms were defined over the same 0–24 h interval, this evaluation benchmarks early empiric management as a stewardship-oriented process measure rather than a strict admission-time (minute-0) bedside decision.

Building on this clinical framework, LLM recommendations were generated via the OpenAI API (official python package version 2.17.0) [50] using the Responses endpoint (model identifier returned by the API at execution time: gpt-5.2). We used a fixed prompt template populated with the aforementioned structured patient context and the local epidemiology module. Recommendations were produced post hoc and did not influence care. Requests were executed between 2026-02-07T13:23:14Z and 2026-02-07T13:39:43Z. We utilized temperature = 0.0 and max_output_tokens = 800, with up to three retry attempts for transient failures. For privacy, we set store = false in API requests. All other generation parameters were left at API defaults (e.g., top_*p* = 1.0; presence_penalty = 0; frequency_penalty = 0).

Outputs were constrained to a structured format (validated against a strict JSON schema) mapped to a predefined antibiotic dictionary to enable deterministic downstream scoring and auditing. Model calls were audit-logged (request/response metadata, timestamps, attempt counters, and response identifiers) and integrity-protected using cryptographic hashing (SHA-256) with a versioned manifest enabling reconstruction of the reported aggregate results. The public audit bundle provides the prompt template with placeholders only, output constraints, allowed regimen code list, JSON schemas, mapping tables, hashed manifests, and derived summary outputs sufficient to reproduce all reported statistics; raw per-admission prompts and full model responses remain restricted due to privacy and institutional governance. The full prompt template and structured-output specification are provided in the Supplementary Materials (Supplementary File S1). Evaluation/reporting practices were informed by early-stage clinical AI guidance and LLM-specific reporting considerations [24,25].

### 4.6. Outcomes (Contextual Stewardship Guardrails)

#### 4.6.1. Regimen Definition (24 h)

For each admission, the clinician empiric regimen was defined as the set of systemic antibacterial agents initiated concurrently as the initial empiric order set within the first 24 h of admission (0–24 h empiric management window), as verified using pharmacy dispensing request timestamps. The LLM regimen was defined as the antibacterial agents recommended by the LLM for the same 24 h empiric decision point, normalized through the same antibiotic dictionary/mapping.

#### 4.6.2. Primary (Prespecified) Endpoint

The primary endpoint was any contextual guardrail violation (paired binary), defined as the presence of ≥1 unjustified broad-spectrum element within the 24 h empiric regimen under pre-specified structured context rules.

Rationale, provenance, and intended role of the contextual guardrails. The contextual guardrails were defined a priori as a research operationalization of core antimicrobial stewardship principles, not as an externally validated appropriateness score. These guardrails were defined by the study team for benchmarking purposes and were not intended to represent a hospital prescribing protocol or a formal institutional stewardship policy. Their purpose was to reduce arbitrariness in judging contextual adequacy in retrospective empiric prescribing by focusing on a small number of broad-spectrum escalation decisions with disproportionate stewardship relevance: carbapenem use, antipseudomonal use, and anti-MRSA use. These classes were selected because they represent high-leverage empiric choices repeatedly emphasized in stewardship guidance for risk-stratified initiation, indication review, and early de-escalation, and because they capture a substantial share of potentially avoidable spectrum expansion within the first 24 h of admission [13,16,17,18]. Specifically, the selection of these three escalation domains was informed by international stewardship guidance (IDSA/SHEA implementation guidelines [27], ATS/IDSA pneumonia guidelines [18], IDSA complicated UTI guidelines [19]), WHO AWaRe classification principles [15], and published carbapenem-sparing frameworks [16]. Accordingly, the guardrails were intended as an auditable admission-level benchmarking construct for contextual broad-spectrum escalation rather than as a universal measure of overall prescribing appropriateness.

The operational logic of the guardrails was literature-anchored and translated into prespecified binary justification proxies available in the retrospective dataset. In practical terms, the framework asked whether use of these broad-spectrum classes was supported by structured early severity signals, MDR-risk/history markers, or class-specific microbiological risk proxies captured at admission-level abstraction. This approach was chosen to standardize benchmarking across admissions and between study arms under the same structured context. The resulting framework was designed for internal auditability and paired comparison, not as a formally externally validated stewardship score; therefore, its results should be interpreted as benchmarking within a predefined rule-based construct rather than as definitive adjudication of optimal care.

Contextual guardrail violations and penalty. For each admission and each arm (clinician vs. LLM), we evaluated three prespecified contextual guardrail components: carbapenem use, antipseudomonal use, and anti-MRSA use. A component violation was defined as antibiotic class use in the absence of prespecified justification proxies (binary rules), yielding component indicators *I_k*. The contextual guardrail penalty was computed as a weighted sum of component violations:

(1) $$\mathrm{Penalty}=\sum_{k}w_{k}\;I_{k},$$

where k ∈ {carb, APS, MRSA}, *I_k* = 1 if component k is violated (i.e., the class is used without a prespecified justification proxy) and 0 otherwise. The prespecified weights were *w*_carb = 2, *w*_APS = 1, and *w*_MRSA = 1; equivalently, Penalty = 2·*I*_carb + 1·*I*_APS + 1·*I*_MRSA. The paired delta was defined as ΔPenalty = Penalty (LLM)—Penalty (clinician) (negative values favor the LLM arm). Full component definitions (trigger logic, data fields, mappings, and weights) are provided in Supplementary Table S3.

The weighting scheme was prespecified as a pragmatic hierarchy of unjustified spectrum expansion rather than a validated utility scale. Unjustified carbapenem use received greater weight because carbapenems occupy a particularly high-leverage position in stewardship and carbapenem-sparing strategies, whereas unjustified antipseudomonal and anti-MRSA additions were treated as single-step broad-spectrum escalations of comparable ordinal severity [16]. The weighted penalty was therefore intended to preserve gradation in contextual breadth beyond a simple binary violation indicator, not to quantify clinical harm or ecological damage on an interval scale.

Justification proxies used in guardrails. To operationalize contextual justification in a reproducible way, we prespecified a limited set of binary proxy domains available in the structured retrospective dataset. Severity proxy (SEVERE) was defined using early clinical indicators within the first 24 h (ICU admission/transfer, septic shock documentation, vasopressor use, mechanical ventilation, or respiratory failure documentation). Multidrug-resistant risk proxy (MDR_RISK) was defined using prespecified history/setting variables (prior ESBL/CRE/VRE, systemic antibiotic exposure in the prior 90 days, hospitalization in the prior 90 days, long-term care facility residence, or healthcare-associated acquisition setting). Anti-MRSA justification was defined by documented prior MRSA colonization. These proxies were not intended to exhaust bedside clinical judgment, but to create a structured and auditable rule set applicable to both the clinician and LLM regimens under the same recorded admission context. Exact Boolean logic, field names, stewardship rationale, and guideline-level anchor sources are summarized in Supplementary Table S3.

#### 4.6.3. Secondary/Supplementary Endpoints (Multiplicity Caution)

Secondary and supplementary analyses were prespecified as non-confirmatory and interpreted with caution regarding multiplicity. These included:

- empiric antibiotic cost differences at 24 h (N = 493) and at 72 h only in admissions where clinician empiric therapy was continued for ≥3 days (N = 323);
- ΔDDD/24 h (N = 493) calculated using the WHO ATC/DDD methodology [51];
- microbiology-evaluable coverage analyses (paired; N = 158; descriptive due to selection);
- regimen concordance measures;
- AWaRe class distributions (supplementary; interpretation-sensitive) based on the WHO AWaRe framework [15];
- exploratory multivariable models (complete-case; N = 493), reported as exploratory only

Microbiology-evaluable admissions were defined as admissions with ≥1 clinical culture collected within the first 24 h of admission (collection timestamp) that yielded an index organism with interpretable antimicrobial susceptibility results, and that could be plausibly linked to the presumed infection syndrome used for empiric therapy assessment. Paired evaluability required that active-coverage classification be possible in both arms; admissions not meeting these criteria in either arm were coded as non-evaluable (not missing) and excluded from the paired coverage analysis. Because culture collection/positivity and susceptibility availability are post-baseline clinical processes correlated with severity and clinical trajectory, microbiology-evaluable analyses are interpreted as subset-level exploratory process benchmarking and are inherently susceptible to selection bias.

AWaRe-derived delta metrics (Supplementary Data). For regimen-level AWaRe summarization, we derived two paired metrics: Δ AWaRe mean score and Δ AWaRe max score (LLM − clinician), computed from the regimen’s agent-level AWaRe mapping (see Table S10a–c). These metrics are reported as supplementary paired-delta outcomes and included in the prespecified paired-delta multiplicity family (Table S12).

### 4.7. Statistical Analysis

Analyses followed a pre-specified hierarchy: (1) primary endpoint tested first; (2) key secondary endpoint interpreted as prespecified conditional on primary significance; all other endpoints were treated as secondary/supplementary/exploratory with explicit multiplicity caution. Missing data were handled using a complete-case approach for each analysis; denominators therefore reflect admissions with non-missing outcome data in both paired arms (and, where applicable, membership in prespecified subsets). No imputation was performed. Regarding the paired primary and key secondary endpoints, all included admissions had complete endpoint data (N = 493). For endpoints defined on restricted subsets (e.g., 72 h continuation, microbiology-evaluable subset), analyses were performed only in admissions meeting the predefined evaluability criteria, with denominators reported explicitly.

#### 4.7.1. Primary Endpoint (Paired Binary)

For the paired binary primary endpoint, we used the exact McNemar test based on discordant pairs. We report the paired 2 × 2 table (n00, n01, n10, n11), the paired risk difference (RD, LLM − Clin), and a matched odds ratio estimated from discordant pairs using a continuity correction: OR = (n01 + 0.5)/(n10 + 0.5). A 95% confidence interval was computed using a Wald interval on the log scale: log(OR) ± 1.96 × √(1/(n01 + 0.5) + 1/(n10 + 0.5)).

#### 4.7.2. Key Secondary Endpoint (Paired Integer Deltas with Many Ties)

For Δ contextual guardrail penalty (LLM − Clin), we report median and IQR, the number of ties, and compare paired deltas using the Wilcoxon signed-rank test and a sign test on non-tied pairs (appropriate when ties are frequent) [52]. We additionally computed a bootstrap 95% confidence interval for the median Δ by resampling admissions with replacement (n_boot = 5000, seed = 7) [53].

#### 4.7.3. Secondary Analyses and Exploratory Modeling

Secondary paired continuous outcomes (e.g., cost deltas, ΔDDD/24 h) were analyzed using paired nonparametric methods (Wilcoxon signed-rank), reporting denominators per module; when ties were frequent, we additionally report a sign test on non-tied pairs (prespecified for Δ contextual guardrail penalty and reported as a sensitivity analysis for other paired-delta endpoints); Wilcoxon signed-rank tests use the standard handling of zero differences per the implementation. DDD calculations followed WHO ATC/DDD guidance [51]. To provide transparent multiplicity control across the prespecified paired-delta family (m = 6), we report Holm step-down adjusted *p*-values in Supplementary Table S12 for: Δ contextual guardrail penalty (key secondary), Δ empiric antibiotic cost at 24 h, Δ empiric antibiotic cost at 72 h (continued-therapy subset), Δ AWaRe mean score, Δ AWaRe max score (derived from regimen-level AWaRe mapping; see Table S10), and Δ DDD/24 h (all two-sided). Unadjusted *p*-values are reported alongside each endpoint (Table 2 for main-text endpoints; Supplementary Tables S1–S12 for supplementary endpoints). Inference for the prespecified endpoint hierarchy followed the prespecified strategy (primary tested first; key secondary interpreted conditional on primary). Holm-adjusted *p*-values are reported for the prespecified paired-delta family for transparency but are not used to re-define the primary/key-secondary hierarchy. Where an outcome is defined on a prespecified subset (e.g., 72 h cost), the corresponding *p*-value is computed on that subset denominator and then included in the Holm family. Microbiology-evaluable coverage analyses were treated as subset-level exploratory process benchmarking due to selection into the evaluable subset. The microbiology-evaluable paired subset was defined as admissions with an identified index organism and interpretable susceptibility allowing classification of active empiric coverage in both arms; all other admissions were classified as non-evaluable. Because culture acquisition/positivity are post-baseline processes correlated with severity and clinical trajectory, analyses in this subset are interpreted as process benchmarking rather than causal effectiveness. Selection-bias QC comparing evaluable vs. non-evaluable admissions is reported in Supplementary Table S2.

Exploratory multivariable models were conducted as complete-case analyses (N = 493) and reported as exploratory only, with attention to instability/separation risks typical in sparse paired events.

For structured variables defining guardrail triggers (e.g., severe sepsis criteria, comorbidities), the absence of documentation in the electronic health record was pragmatically treated as the absence of the condition, reflecting standard clinical prescribing constraints under uncertainty.

Empiric antibiotic costs were estimated as drug acquisition costs only, using a fixed unit-price mapping applied to standardized regimen codes; administration, monitoring, and hospitalization costs were not included. Unit prices (including price year/source) and the EUR conversion assumption are specified in Supplementary Data File S2 (“data_file_s2_costing”); costs therefore reflect drug acquisition only, without administration/monitoring or downstream costs.

#### 4.7.4. Software and Reproducibility

All analyses were conducted using a fully scripted and version-controlled pipeline implemented in Python (version 3.14.2), with pandas for data manipulation, NumPy (version 2.4.1) and SciPy (version 1.17.0) for numerical operations, and statsmodels (version 0.14.6) for statistical modeling. Analysis scripts, data dictionaries, antibiotic code-mapping tables, JSON schemas, and non-identifiable audit artifacts (including file manifests and cryptographic checksums) are publicly available in a versioned repository (Zenodo: https://doi.org/10.5281/zenodo.18731938).

### 4.8. Ethics, Data Governance, and Availability Statements

The study protocol was reviewed and approved by the Ethics Committee of the Clinical Emergency Hospital of Bucharest, approval no. 5352, issued on 2 July 2025, prior to data analysis. The study involved no direct patient contact and no modification of standard clinical care. Data processing and storage followed applicable data protection requirements (GDPR) [54].

All analyses were conducted using a fully scripted and auditable pipeline. A complete reproducibility package including analysis code, data dictionaries, code-mapping tables, JSON schemas, derived aggregate tables, and audit artifacts (e.g., hashes and file manifests), is publicly available at https://doi.org/10.5281/zenodo.18731938.

Patient-level EHR-derived data and raw per-admission LLM prompt/response payloads are not publicly shared due to privacy, legal (GDPR), and institutional governance constraints. Access to de-identified patient-level data or to a controlled analysis environment may be considered upon reasonable request, subject to institutional approvals, a data-sharing agreement, and applicable data-protection regulations.

Regarding the LLM input privacy safeguards, no direct identifiers were transmitted to the API. All admissions were processed using pseudonymous study identifiers. Prompts contained only de-identified, structured clinical variables (e.g., age, sex, coded comorbidities, syndrome labels, and selected admission laboratory values). We strictly excluded direct identifiers (names, national identifiers, addresses), exact calendar dates, and free-text clinical notes; temporal information was provided only in relative or coarse form where needed for clinical interpretation. Data were processed in a controlled environment with access restrictions, API requests were sent with store = false, and all payloads were retained locally in an auditable log. Consistent with the vendor’s policy for business/API services, inputs and outputs are not used for model training by default, unless an organization explicitly opts in; retention behavior (e.g., abuse-monitoring logs and application-state retention) follows the platform’s documented data controls [55].

## 5. Conclusions

In this single-center retrospective shadow-mode paired evaluation of 493 admissions, LLM-generated empiric antibiotic regimens showed greater concordance with a pre-specified stewardship benchmarking framework than clinician regimens, with fewer contextual broad-spectrum escalations and modestly lower 24 h acquisition costs. These findings should not be interpreted as evidence of clinical superiority, patient safety, or causal effectiveness. Rather, they represent process-level benchmarking within a rule-based stewardship construct and are best understood as an early pre-implementation step for assessing alignment and identifying potential failure modes without influencing patient care. Prospective shadow-mode validation followed by a human-in-the-loop stewardship trial with pre-registered endpoints and governance safeguards is warranted to assess whether any such process-level signal translates into patient-centered benefit or harm. No patient-centered outcomes were evaluated, and no causal conclusions about clinical benefit or harm can be drawn from this study.

## Figures and Tables

**Table 1:** Baseline characteristics of the paired admission cohort (N = 493).

Characteristic	Total (N = 493)
**Demographics**	
Age, years, median (IQR)	72 (64–82)
Female sex, n (%)	250 (50.7%)
Length of stay, days, median (IQR)	9 (5–15)
**Study period**	
Cohort year 2020, n (%)	98 (19.9%)
Cohort year 2021, n (%)	100 (20.3%)
Cohort year 2022, n (%)	100 (20.3%)
Cohort year 2023, n (%)	98 (19.9%)
Cohort year 2024, n (%)	97 (19.7%)
**Acquisition setting**	
Community-onset, n (%)	459 (93.1%)
Healthcare-associated, n (%)	34 (6.9%)
**Index syndrome (top categories)**	
Community-acquired pneumonia, n (%)	311 (63.1%)
Urinary tract infection—unspecified site, n (%)	56 (11.4%)
Bloodstream infection/sepsis, n (%)	50 (10.1%)
COPD infectious exacerbation, n (%)	32 (6.5%)
Urinary tract infection—pyelonephritis, n (%)	29 (5.9%)
Skin and soft-tissue infection, n (%)	7 (1.4%)
Other syndromes, n (%)	8 (1.6%)
**Severity/support within 24 h**	
Sepsis documented, n (%)	183 (37.1%)
Septic shock documented, n (%)	129 (26.2%)
Respiratory failure documented, n (%)	374 (75.9%)
Mechanical ventilation, n (%)	147 (29.8%)
Vasopressors, n (%)	127 (25.8%)
ICU transfer, n (%)	141 (28.6%)
**Prior exposure/MDR-risk proxies**	
Antibiotics in prior 90 days, n (%)	75 (15.2%)
Hospitalization in prior 90 days, n (%)	70 (14.2%)
Long-term care facility resident, n (%)	32 (6.5%)
Prior MRSA colonization/infection, n (%)	6 (1.2%)
Prior ESBL/CRE/VRE history, n (%)	22 (4.5%)
Home antibiotics before admission: Yes, n (%)	65 (13.2%)
Home antibiotics before admission: No, n (%)	428 (86.8%)
**Comorbidities**	
Diabetes mellitus, n (%)	160 (32.5%)
COPD, n (%)	94 (19.1%)
Chronic kidney disease, n (%)	137 (27.8%)
Congestive heart failure, n (%)	241 (48.9%)
Atrial fibrillation, n (%)	180 (36.5%)

Values are n (%) unless otherwise stated. Severity/support flags refer to the first 24 h after admission. All years reflect the final counts after applying the deduplication rule (first admission per patient). Abbreviations: IQR, interquartile range; ICU, intensive care unit; MDR, multidrug-resistant.

**Table 2:** Primary, key secondary, and selected secondary endpoints (paired, first 24 h window).

Panel	Endpoint	N	Clinician	LLM	Discordant Pairs	Effect	*p* Value	Median 95% CI (Bootstrap)
A (prespecified)	Primary: Any contextual guardrail violation (composite)	493	84/493 (17.0%)	24/493 (4.9%)	Clin = 1/LLM = 0: 76; Clin = 0/LLM = 1: 16	Matched OR 0.216 (95% CI 0.127–0.367) RD (LLM − Clin) −0.122	*p* = 1.60 × 10^−10^	
B (prespecified)	Key secondary: Δ contextual guardrail penalty (LLM − Clin)	493	—	—	Δ < 0 (lower): 79; Δ > 0 (higher): 16; Δ = 0: 398	Median 0 (IQR 0–0) Mean −0.219 (SD 0.789)	Wilcoxon *p* = 9.48 × 10^−11^; Sign *p* = 3.47 × 10^−11^	Median 0 (IQR 0–0); 95% CI 0–0
C (Secondary; multiplicity caution)	Any broad-spectrum class used (carb OR APS OR anti-MRSA)	493	289/493 (58.6%)	195/493 (39.6%)	Clin = 1/LLM = 0: 143; Clin = 0/LLM = 1: 49	Matched OR 0.345 (95% CI 0.250–0.477) RD (LLM − Clin) −0.191	*p* = 7.26 × 10^−12^	
C (Secondary; multiplicity caution)	Carbapenem contextual violation	493	39/493 (7.9%)	9/493 (1.8%)	Clin = 1/LLM = 0: 36; Clin = 0/LLM = 1: 6	Matched OR 0.178 (95% CI 0.077–0.410) RD (LLM − Clin) −0.061	*p* = 2.83 × 10^−6^	
C (Secondary; multiplicity caution)	Antipseudomonal contextual violation	493	31/493 (6.3%)	2/493 (0.4%)	Clin = 1/LLM = 0: 30; Clin = 0/LLM = 1: 1	Matched OR 0.049 (95% CI 0.010–0.253) RD (LLM − Clin) −0.059	*p* = 2.98 × 10^−8^	
C (Secondary; multiplicity caution)	Anti-MRSA contextual violation	493	36/493 (7.3%)	17/493 (3.4%)	Clin = 1/LLM = 0: 32; Clin = 0/LLM = 1: 13	Matched OR 0.415 (95% CI 0.220–0.784) RD (LLM − Clin) −0.039	*p* = 6.61 × 10^−3^	
C (Secondary; multiplicity caution)	Δ empiric antibiotic cost, 24 h (EUR; LLM − Clin)	493	—	—	Δ < 0 (lower): 268; Δ > 0 (higher): 164; Δ = 0: 61	Median −1.43 (EUR) (IQR −9.80–0.57) Mean −4.11 (EUR) (SD 14.35)	Wilcoxon *p* = 2.80 × 10^−13^; Sign *p* = 6.41 × 10^−7^	Median 95% CI (bootstrap): −4.09 to −0.07

Notes: Unit of analysis is the admission; all comparisons are paired within admission (clinician vs. LLM; N = 493). For paired binary endpoints, we report arm-specific counts (%), discordant pairs (Clinician = 1/LLM = 0 and Clinician = 0/LLM = 1), matched odds ratios (0.5 continuity correction, as prespecified) with Wald 95% CIs on the log scale, risk differences (LLM − Clinician), and exact McNemar *p*-values. For paired deltas, Δ denotes LLM − clinician; negative Δ indicates a lower value in the LLM arm (e.g., lower contextual penalty or lower empiric cost). Panel A is the prespecified primary endpoint; Panel B is the prespecified key secondary endpoint; Panel C endpoints are secondary and should be interpreted with multiplicity caution. Holm-adjusted *p*-values for the prespecified paired-delta family are provided in Supplementary Table S12. Broad-spectrum class exposure is defined as any carbapenem, antipseudomonal β-lactam, or anti-MRSA agent used during the first 24 h empiric management window. Abbreviations: LLM, large language model; OR, odds ratio; CI, confidence interval; RD, risk difference; IQR, interquartile range; SD, standard deviation; MRSA, methicillin-resistant *Staphylococcus aureus*; EUR, euros.

## Data Availability

Derived, non-identifiable audit artifacts (analysis code, schemas, mapping tables, derived summary tables, and file manifests with SHA-256 checksums) are available at https://doi.org/10.5281/zenodo.18731938. The public audit bundle includes the fully scripted statistical analysis pipeline, antibiotic code-mapping tables, epidemiology/QC scripts, derived analysis outputs, and file manifests enabling integrity verification. With the exception of a limited de-identified case-level QC table for the five NO_ANTIBIOTIC admissions (Supplementary Table S4), individual-level EHR-derived data and per-admission LLM payloads remain restricted due to institutional governance and GDPR.

## Supplementary Materials

The following supporting information can be downloaded at: https://www.mdpi.com/article/10.3390/antibiotics15040368/s1, Table S1 (Cohort flow/inclusion–exclusion decision counts); Table S2 (Microbiology-evaluable subset: selection-bias QC, evaluable vs. non-evaluable); Table S3 (Contextual guardrail rule specification, including trigger logic, variable mapping, penalty weights, stewardship rationale, and anchor references); Table S4 (NO_ANTIBIOTIC cases: case-level QC summary); Table S5 (Predictors of nonzero Δ guardrail penalty; multivariable logit with robust SE); Table S5a (Predictors of LLM improvement in contextual guardrails; multivariable logit with robust SE); Table S6 (Missingness map for baseline covariates and endpoint-defining variables; n missing and % missing per variable; includes not-applicable/nonevaluable where subset-defined); Table S6a (Missingness and complete-case QC); Table S7 (Regimen concordance summary); Table S8 (Microbiology-evaluable composite: active coverage AND no contextual violation); Table S9 (Contextual guardrail component endpoints); Table S10a–c (AWaRe binaries, transitions, and unmapped QC); Table S11 (Low-risk subgroup analyses); data_file_S1 (De-identified screening log for chart-reviewed candidate admissions; includes decision codes and year); Table S12 (Holm step-down adjustment across the prespecified paired-delta family, m = 6; includes the key secondary Δ penalty plus five additional paired-delta outcomes; two-sided tests); data_file_s2_costing (Costing assumptions—drug acquisition only); Supplementary Text S1 (full prompt template—placeholders only—output constraints, allowed regimen code list, and strict response format); Table S13 (Regimen concordance between clinician and LLM regimens).

## Abbreviations

The following abbreviations are used in this manuscript:

AI	Artificial intelligence
AMR	Antimicrobial resistance
AMS	Antimicrobial stewardship
APS	Antipseudomonal broad-spectrum β-lactam
ASP	Antimicrobial stewardship program
AWaRe	Access, Watch, Reserve (WHO antibiotic classification)
CDI	*Clostridioides difficile* infection
CI	Confidence interval
DDD	Defined daily dose
EHR	Electronic health record
ICU	Intensive care unit
IQR	Interquartile range
LLM	Large language model
MDR	Multidrug-resistant
MRSA	Methicillin-resistant *Staphylococcus aureus*
ESBL	Extended-spectrum beta-lactamase
CRE	Carbapenem-resistant *Enterobacterales*
VRE	Vancomycin-resistant *Enterococci*
RD	Risk difference
