# Supplementary material for: Large Language Model Recommendations for Empiric Antibiotics Versus Clinician Prescribing: A Non-Interventional Paired Retrospective Antimicrobial Stewardship Analysis
Source: Antibiotics (Basel). 2026 Apr 2;15(4):368. doi: 10.3390/antibiotics15040368 (PMC13113701; doi:10.3390/antibiotics15040368)
Supplement: Supplementary file 1 [file antibiotics-15-00368-s001.zip › Table_S13.pdf]

Table . Regimen concordance between clinician and LLM regimens (N = 493). Overlap defined as ≥1 identical antibiotic agent present in both regimen sets (molecule-level mapping)

Metric	N	%
Exact identical regimen set	57	11.6%
Same primary agent (first-listed antibiotic)	137	27.8%
Any overlap (≥1 shared agent)	148	30.0%
No overlap (0 shared agents)	345	70.0%
