# Supplementary material for: Large Language Model Recommendations for Empiric Antibiotics Versus Clinician Prescribing: A Non-Interventional Paired Retrospective Antimicrobial Stewardship Analysis
Source: Antibiotics (Basel). 2026 Apr 2;15(4):368. doi: 10.3390/antibiotics15040368 (PMC13113701; doi:10.3390/antibiotics15040368)
Supplement: Supplementary file 1 [file antibiotics-15-00368-s001.zip › Table_S1a.pdf]

Table 1. Baseline characteristics of the paired admission cohort (N = 493).

Characteristic	Total (N = 493)
**Demographics**	
Age, years, median (IQR)	72 (64–82)
Female sex, n (%)	250 (50.7%)
Length of stay, days, median (IQR)	9 (5–15)
**Study period**	
Cohort year 2020, n (%)	98 (19.9%)
Cohort year 2021, n (%)	100 (20.3%)
Cohort year 2022, n (%)	100 (20.3%)
Cohort year 2023, n (%)	98 (19.9%)
Cohort year 2024, n (%)	97 (19.7%)
**Acquisition setting**	
Community-onset, n (%)	459 (93.1%)
Healthcare-associated, n (%)	34 (6.9%)
**Index syndrome (top categories)**	
Community-acquired pneumonia, n (%)	311 (63.1%)
Urinary tract infection - unspecified site, n (%)	56 (11.4%)
Bloodstream infection / sepsis, n (%)	50 (10.1%)
COPD infectious exacerbation, n (%)	32 (6.5%)
Urinary tract infection – pyelonephritis, n (%)	29 (5.9%)
Skin and soft-tissue infection, n (%)	7 (1.4%)
Other syndromes, n (%)	8 (1.6%)
**Severity / support within 24 h**	
Sepsis documented, n (%)	183 (37.1%)
Septic shock documented, n (%)	129 (26.2%)
Respiratory failure documented, n (%)	374 (75.9%)
Mechanical ventilation, n (%)	147 (29.8%)
Vasopressors, n (%)	127 (25.8%)
ICU transfer, n (%)	141 (28.6%)
**Prior exposure / MDR-risk proxies**	
Antibiotics in prior 90 days, n (%)	75 (15.2%)
Hospitalization in prior 90 days, n (%)	70 (14.2%)
Long-term care facility resident, n (%)	32 (6.5%)
Prior MRSA colonization/infection, n (%)	6 (1.2%)
Prior ESBL/CRE/VRE history, n (%)	22 (4.5%)
Home antibiotics before admission: Yes, n (%)	65 (13.2%)
Home antibiotics before admission: No, n (%)	428 (86.8%)
**Comorbidities**	
Hypertension, n (%)	374 (75.9%)
Diabetes mellitus, n (%)	160 (32.5%)
COPD, n (%)	94 (19.1%)
Chronic kidney disease, n (%)	137 (27.8%)
Congestive heart failure, n (%)	241 (48.9%)

Atrial fibrillation, n (%)	180 (36.5%)
Prior stroke, n (%)	98 (19.9%)
Cirrhosis, n (%)	27 (5.5%)
Malignancy, n (%)	90 (18.3%)
Immunosuppression, n (%)	29 (5.9%)

Values are n (%) unless otherwise stated. IQR, interquartile range. Severity/support flags refer to the first 24 h after admission. All years reflect the final counts after applying the deduplication rule (first admission per patient)
